# Selecting a Window Size for Phylogenomic Analyses of Whole Genome Alignments Using AIC

**DOI:** 10.1093/sysbio/syaf053

**Published:** 2025-08-06

**Authors:** Jeremias Ivan, Paul Frandsen, Robert Lanfear

**Affiliations:** Research School of Biology, Australian National University, Canberra, ACT 2601, Australia; Department of Plant and Wildlife Sciences, Brigham Young University, Provo, UT 846002, USA; Research School of Biology, Australian National University, Canberra, ACT 2601, Australia

**Keywords:** Concatenation, gene tree discordance, gene tree estimation error, simulation, stepwise approach

## Abstract

Gene tree discordance along a set of aligned genomes presents a challenge for phylogenomic methods to identify the non-recombining regions and reconstruct the phylogenetic tree for each region. To address this problem, many studies used the non-overlapping window approach, often with an arbitrary selection of fixed window sizes that potentially include intra-window recombination events. In this study, we propose an information theoretic approach to select a window size that best reflects the underlying histories of the alignment. First, we simulated chromosome alignments that reflected the key characteristics of an empirical data set and found that the Akaike information criterion (AIC) is a good predictor of window size accuracy in correctly recovering the tree topologies of the alignment. To address the issue of missing data in empirical data sets, we designed a stepwise non-overlapping window approach that compares the AIC of two window sizes at a time, retaining only genomic regions that can be analyzed using both window sizes. We then applied this method to the genomes of *Heliconius* butterflies and great apes. We found that the best window sizes for the butterflies’ chromosomes ranged from ≤125 to 250 bp, which are much shorter than those used in a previous study even though this difference in window size did not significantly change the most common topologies across the genome. On the other hand, the best window sizes for great apes’ chromosomes ranged from 500 bp to 1 kb with the proportion of the major topology (grouping human and chimpanzee) falling between 60% and 87%, consistent with previous findings. Additionally, we observed a notable impact of gene tree estimation error and concatenation when using small and large windows, respectively. For instance, the proportion of the major topology for great apes was 50% when using 250 bp windows, but reached almost 100% for 64 kb windows. In conclusion, our study highlights the challenges associated with selecting a fixed window size in non-overlapping window analyses and proposes the AIC as a less arbitrary way to select the optimal window size when running the non-overlapping window method on whole genome alignments.

Most phylogenomic studies begin by sequencing and assembling the entire genomes of multiple individuals and/or species, which—when coming from closely related species—can typically be aligned to one another almost in their entirety. The next logical step is to then reconstruct the evolutionary history of every site in those aligned genomes, which can reveal important information about the group, such as demographic history and introgression events (e.g., Pease et al. 2016; Copetti et al. 2017; Morales-Briones et al. 2021; Lescroart et al. 2023; McLay et al. 2023; Herrig et al. 2024). However, as different genomic regions within the alignment may follow distinct evolutionary histories, this variation often leads to topological incongruence across the genomes (Maddison 1997; Degnan and Rosenberg 2009; Mallet et al. 2016; Hibbins et al. 2023; Steenwyk et al. 2023), making their estimation particularly challenging.

A number of approaches have been proposed to reconstruct the evolutionary histories along a set of aligned genomes. Population genetic-based methods that use ancestral recombination graph (ARG) data structures (e.g., ARGweaver [Rasmussen et al. 2014], tsinfer [Kelleher et al. 2019], and Relate [Speidel et al. 2019]) represent a powerful approach to estimate the evolutionary history of every site in the genome. However, these methods have considerable challenges, including the trade-offs between accuracy and scalability (Brandt et al. 2024). At deeper timescales, hidden Markov models (HMMs) can be used to co-infer the history of every site in the genome as well as the recombination breakpoints in the presence of extensive incomplete lineage sorting (ILS), recombination, and hybridization, with an assumption that neighboring sites are likely to share evolutionary histories. However, the current implementations of HMMs do not scale to whole genome sequence analysis (e.g., PhyML_Multi [Boussau et al. 2009] and PhyloNet-HMM [Liu et al. 2014]) or are limited to rooted triplets (e.g., Coal-HMM [Hobolth et al. 2007; Dutheil et al. 2009]). Recently, Wong et al. (2024) proposed the Mixture Across Sites and Trees (MAST) model that uses a mixture of bifurcating trees to infer the weight of each topology (i.e., tree without branch lengths) within the genomic sequences. This model determines the likelihood that each site in the genome follows a particular history, and can scale to large data sets. However, MAST requires a set of input topologies that needs to be derived from other methods. Alternatively, many phylogenomic studies used only a small subset of the genomes, such as exons or introns, to infer the evolutionary histories of the group (e.g., Chen et al. 2017; Vanderpool et al. 2020). Although this approach is particularly powerful at deeper timescales where aligning the entire genomes is impractical or impossible, it also has its own challenges. For example, there is growing evidence that evolutionary histories can vary even within relatively short genomic regions (Scornavacca and Galtier 2017; Mendes et al. 2019), and that non-recombining regions (sometimes called “coalescent-genes” or “c-genes”) might be surprisingly small even in data sets with modest recombination rates (e.g., ∼12 bp in mammals, although this remains an active area of discussion [Edwards et al. 2016; Springer and Gatesy 2016, 2018]). The upshot is that inferring the histories of every site in a set of aligned genomes remains very challenging.

One popular and relatively simple approach to the problem is to first partition the genome alignment into non-overlapping blocks of equal size called “windows.” Then, a phylogenetic tree is reconstructed for every window with the assumption that each window has no intra-window recombination (i.e., each window would have only one substitution model and one tree [one history] that best explain the alignment). This approach has been applied by many studies to explore the variation of phylogenetic histories across genomes (e.g., Fontaine et al. 2015; Pease et al. 2016; Edelman et al. 2019; Meleshko et al. 2021; Yang et al. 2021; Feng et al. 2023; Lescroart et al. 2023; Herrig et al. 2024). However, one of the main challenges of this approach is that there is no objective method to determine the ideal window size (i.e., the window size that most closely reflects the recombination patterns of the alignment). For example, Yang et al. (2021) assessed the genome-wide topology distribution of Mediterranean lizards using six different window sizes, which ranged from 5 to 200 kb. On the other hand, Herrig et al. (2024) used longer windows of 10 kb, 50 kb, 100 kb, 500 kb, and 1 Mb to generate the species tree of *Neodiprion* sawflies. Despite this wide array of choices of window size, there remains no objective justification for the choice of any particular size.

This may not be an issue if the selected window size is sufficiently short that most windows do not contain recombination breakpoints. However, the empirical evidence suggests that this is often not the case. For example, Edelman et al. (2019) found that in the overwhelming majority of cases, the tree topology changed from one window to the next. This implies that many windows were likely to contain breakpoints (in this case mainly due to introgression), because if the windows contained no breakpoints, then many consecutive windows would be expected to have identical tree topologies resulting from the splitting of a long-conserved region into many windows. Because non-overlapping windows are widely used and the optimal window size is difficult to determine *a priori*, a less arbitrary approach to select the window size would provide a good starting point to generate a set of window alignments and their associated gene trees. However, gene tree estimation error remains a challenge for the non-overlapping window approach. Once a window size is selected, any method used to estimate the phylogenetic tree for every window (e.g., Neighbor Joining, Maximum Likelihood [ML], or Bayesian methods) must make those estimations using potentially very limited information from the sequence (Gatesy and Springer 2014). In order to further refine the estimates of the gene trees, other methods such as Espalier (Rasmussen and Guo 2023), MAST (Wong et al. 2024), and BUCKy (Larget et al. 2010) can be used.

In this study, we sought to develop a less arbitrary window size selection method using information theoretic criteria commonly used in phylogenetics, such as the Akaike information criterion (AIC [Akaike 1974]) and Bayesian information criterion (BIC [Schwarz 1978]). In a phylogenetic context, these measures indicate how well the substitution model and the tree explain the data, while also applying a penalty based on the number of parameters used in the model, such as substitution rates and branch lengths. Thus, the AIC and BIC scores may help to choose the optimal window size because they seek to find the best balance between estimating too many parameters from a genome alignment (i.e., having a window size that is too small) and too few (i.e., having a window size that is too large). For example, the AIC has been used to choose the optimal window size in copy-number alteration analysis on low-coverage tumor sample data (Gusnanto et al. 2014). We acknowledge that a single window size is insufficient to represent an entire genome because recombination rates are well known to vary along the chromosomes (e.g., Jensen-Seaman et al. 2004; McVean et al. 2004; Chan et al. 2012; Edelman et al. 2019). Nevertheless, an information theoretic-informed method to determine a single best-fit window size represents a substantial improvement over the status quo as the window partitions would more closely reflect the recombination patterns of the aligned genomes. In order to achieve this, we first used simulated data to develop an information theoretic approach that allows users to select a window size that balances over- and under-parameterization. We then applied this approach to select the optimal window sizes for empirical data sets of *Heliconius* butterflies and great apes, and used these window sizes to analyze the genome-wide evolutionary histories of these two groups.

## Materials and Methods

### Overview

A non-overlapping window analysis proceeds by choosing a window size, splitting the genome into non-overlapping windows of that size, and estimating a tree for each window, which in this study was done using ML method. In order to ask whether information theoretic criteria (i.e., the AIC and/or BIC) are appropriate to help finding the optimal window size, we simulated a wide range of data sets that mimic a recent empirical study by Edelman et al. (2019) and used these to assess the performance of the AIC and BIC as criteria for choosing the best non-overlapping window size (i.e., the window size with the highest accuracy) when the truth is known. We measured the accuracy of each window size in one of two ways: the proportion of sites from the non-overlapping window analysis assigned to the true (simulated) topology, which we call site accuracy (i.e., a site accuracy of 100% indicates that every site is assigned the topology from which it is simulated), and the root mean squared error (RMSE) of the estimated distribution of tree topologies from the non-overlapping window analysis when compared with the true (simulated) distribution. We assessed the performance of the AIC and BIC by asking whether either correlates with these measures of accuracy. We then applied the best-performing method to two empirical data sets: one from *Heliconius* butterflies (Edelman et al. 2019) and another from great apes (i.e., humans, chimpanzees, gorillas, and orangutans [Waterson et al. 2005; Locke et al. 2011; Scally et al. 2012; Schneider et al. 2017]). All the codes necessary to reproduce the methods in this paper are available at https://github.com/jeremiasivan/SimNOW.

### Simulating Alignments Based on erato-sara Clade of Heliconius Butterflies’ Chromosome

In order to create a realistic simulation scenario, we focused on a well-studied empirical system, the *Heliconius* butterflies. Specifically, we sought to simulate data based on the seven *Heliconius* species that were the primary focus of the work in Edelman et al. (2019): six ingroup species from *erato-sara* clade (*H. erato, H. himera, H. hecalesia, H. telesiphe, H. demeter*, and *H. sara*) and one outgroup species (*H. melpomene*). Throughout, we took care to choose simulation parameters such that the resulting chromosome alignments closely matched the key characteristics of the empirical alignments from Edelman et al. (2019), including the proportion of informative sites and distribution of tree topologies among loci. Full details are provided in the Supplementary Information.

Our simulation design was inspired by Forsythe et al. (2020), except that we used AliSim (Ly-Trong et al. 2022) instead of Seq-Gen (Rambaut and Grass 1997) to simulate the chromosome alignments as AliSim has better runtime and memory usage compared with Seq-Gen and other simulation software (Ly-Trong et al. 2022). We first simulated the gene trees using ms (Hudson 2002), along a species tree with ILS and introgression. Here, the term “gene tree” refers to the local tree topology for each locus generated from ms (even though we did not specifically model the characteristics of a gene, such as exon and intron boundaries, when simulating the data). To do this, we used the species tree topology from Figure 2b of Edelman et al. (2019) and incorporated the three bi-directional introgression events with introgression probabilities estimated from chromosome 11 (chr11) in Thawornwattana et al. (2022): *H. himera*  $ \mathbin{\lower.3ex\hbox{$\buildrel\textstyle\rightarrow\over {\smash{\leftarrow}\vphantom{_{\vbox to.5ex{\vss}}}}$}} $  *H. erato; H. telesiphe*  $\to $  *H. hecalesia*; and *H. telesiphe*  $ \mathbin{\lower.3ex\hbox{$\buildrel\textstyle\rightarrow\over {\smash{\leftarrow}\vphantom{_{\vbox to.5ex{\vss}}}}$}} $ (*H. demeter, H. sara*) (Supplementary Fig. S1). We selected chr11 as the basis of our simulations because it has an intermediate length in the *erato-sara* clade of *Heliconius*’ genomes (i.e., chromosome with intermediate length would have intermediate values for key characteristics of the empirical alignment [e.g., number of sites, recombination rate, and estimated proportions of different gene tree topologies], which provides a good starting point for data simulation). We used speciation dates from Kozak et al. (2015) rather than from Thawornwattana et al. (2022) because the former included an estimate for the outgroup divergence time. Then, we dated each introgression event on the relevant branch proportionally to the estimated time reported in Thawornwattana et al. (2022).

As ms requires the speciation and introgression timings to be in coalescent units, we converted the branch lengths from Myr to coalescent units using estimates of population genetic statistics from *Heliconius* butterflies to provide sensible bounds, and within these bounds selected the parameters that best mimicked the key characteristics of the empirical data set (Supplementary Tables S1 and S2; see Supplementary Information for details). This resulted in using a conversion factor of 4N_e_ generations being equivalent to 0.75 Myr. We set the total alignment length to 10 Mb and varied the recombination rate between 0 (i.e., no recombination) and 2000 (∼126,000 loci with an average length of 80 bp, Supplementary Table S3) using four settings: 0, 20, 200, and 2000. In addition, we examined three degrees of ILS (low, medium, and high) by varying the branch length scaling parameters, as well as one scenario with a medium level of ILS but without an introgression event (see Supplementary Information for details). We simulated 10 replicates per scenario, resulting in 130 sets of simulated chromosomal loci in ms (3 ILS levels × 4 recombination rates × 10 replicates + 10 replicates without introgression). Command lines for each step are provided in the Supplementary Information. Then, for each set of the simulated chromosomal loci, we used the gene trees and the locus lengths as inputs for AliSim (Ly-Trong et al. 2022) to simulate each locus under the Jukes-Cantor (JC; Jukes and Cantor 1969) model, and concatenated them to reconstruct a single 10 Mb chromosome alignment containing all loci.

### Assessing the AIC, BIC, and Accuracy of Non-Overlapping Window Analyses

For each simulated 10 Mb chromosome alignment, we divided the whole alignment into non-overlapping windows with 16 different window sizes, ranging from 100 bp to 10 Mb. At one extreme, a 100 bp window would rarely contain enough phylogenetic signal to build a reliable tree, whereas at the other extreme, a 10 Mb window incorporated the entire simulated chromosome and would violate the assumption that there is only one tree for each window (except for simulations with zero recombination). For each window size, we estimated the gene trees with the JC model using IQ-TREE2 (Minh et al. 2020) with the following command: iqtree2 -S alndir -m JC -blmin 1/window_size -cptime 1000000, where alndir refers to the directory storing all window alignments. The -cptime flag was set to reduce I/O and speed up the analysis. The -blmin flag sets the minimum branch length for all trees to represent at least one substitution per branch, in order to penalize gene tree estimation error in tree topologies from windows with very little phylogenetic information.

We then extracted a single AIC and BIC score (Akaike 1974; Schwarz 1978; Stone 1979) for each window size on each simulated alignment from the IQ-TREE2 output file. Specifically, IQ-TREE2 calculates the AIC score for each window size using the following formula:


\begin{eqnarray*}
\mathrm{ AIC }= - 2{\mathrm{ln}}\left( L \right) + 2k,
\end{eqnarray*}

where $\mathrm{ ln}( L )$ reflects the total log-likelihood across all windows, and $k$ denotes the number of free parameters in the models across all windows (e.g., substitution rates, base frequencies, rate heterogeneity parameters, and branch lengths). IQ-TREE2 calculates the BIC score for each window size using the following formula:


\begin{eqnarray*}
\mathrm{ BIC }= - 2{\mathrm{ln}}\left( L \right) + k \cdot {\mathrm{ln}}\left( n \right),
\end{eqnarray*}

where the additional $n$ parameter represents the number of sites across all windows (i.e., 10 million for our simulations).

We also calculated the accuracy of each of the 16 different window sizes for each simulated alignment by first unrooting the trees from ms using ape v5.6 (Paradis et al. 2004) and then calculated the site accuracy and RMSE for each window size using R v4.2.3 (R Core Team 2023) following these formulas:


\begin{eqnarray*}
{\mathrm{Site\ accuracy}} = \frac{{\mathrm{Sites\ with\ correct\ topology}}}{{\mathrm{Total\ number\ of\ sites}}} \times 100,
\end{eqnarray*}

\begin{eqnarray*}
{\mathrm{ RMSE}} = \sqrt {\sum \frac{{({P}_i - {O}_i)}^2}{n}}.
\end{eqnarray*}

For site accuracy, the numerator counts the number of sites (i.e., individual positions in the alignment) where the estimated gene tree topology from the non-overlapping window analysis matches the true (simulated) gene tree topology from ms at that specific location, whereas the denominator reflects the alignment length (in our case, 10 Mb). For RMSE, ${{P}_i}$ and ${{O}_i}$, respectively, denote the predicted weight (from ms simulation) and observed weight (from non-overlapping window analysis) for the *i*th topology, whereas $n$ denotes the number of unique topologies. We used a binary outcome to measure accuracy (i.e., whether the gene tree topology is exactly right or not) because it is very simple and straightforward to interpret compared with other measures of accuracy (e.g., the number of splits that are shared between the true tree from the simulation and the recovered tree from the non-overlapping window analysis). Moreover, we expect that both site accuracy and RMSE would increase monotonically alongside other measures of accuracy.

### Testing for the Accuracy of the AIC and BIC as Model Selection Criteria for Non-Overlapping Window Analyses

We compared the AIC and BIC scores with our two measures of accuracy (i.e., site accuracy and RMSE) across all window sizes for each simulation. To reflect the way that the AIC and BIC are used, we did this by calculating the loss of accuracy that would be incurred when using each metric to select the best window size (i.e., we compared the accuracy of the most accurate window sizes that give the best site accuracy and/or lowest RMSE with the accuracy of the window size that would be selected using the AIC or BIC).

### Selecting Non-Overlapping Window Sizes on Empirical Data Sets

Our simulation results showed that the AIC, but not the BIC, was a useful metric for selecting non-overlapping window sizes (see Results). Next, we sought to use the AIC to select the optimal window sizes for empirical data sets of *erato*-*sara* clade of *Heliconius* butterflies and great apes. Initially, we applied the method we described above (i.e., calculating the AIC score for a wide range of non-overlapping window sizes and choosing the window size with the best score). However, this approach has a considerable limitation with empirical data sets: the shortest window size that can be analyzed is that for which it is possible to calculate a likelihood for *every* window in the alignment, because comparing AIC scores (in this case between window sizes) assumes that the underlying alignment is identical. As empirical data sets tend to contain many regions where fewer than three species contain data, this can limit the shortest window size that can be analyzed, precluding the calculation of a likelihood in IQ-TREE2, to quite large windows (e.g., many kilobases in the data set we used). Unfortunately, filtering out the gappy positions prior to running a non-overlapping window analysis is discouraged as it would decrease the size of the “true” non-recombining blocks even further, which is likely to increase gene tree estimation error when building the gene trees due to either lack of phylogenetic signal and/or conflicting signals from concatenating two or more non-recombining blocks together.

To overcome this limitation, we designed a stepwise non-overlapping window approach to apply our methods to empirical data sets. In this approach, we start with the longest window size and make a series of pairwise comparisons where the window size is halved each time. Within each pairwise comparison, if a particular window cannot be analyzed due to missing data, we remove that window from the alignment for both window sizes, such that the resulting alignments consist of windows that *can* be analyzed for both members of the pair. This approach allows us to test much smaller window sizes, but at the cost of using smaller proportion of the alignment as the window size becomes smaller. This method is available in the GitHub repository under https://github.com/jeremiasivan/SimNOW/tree/main/empirical_analysis/stepwise_now/.

### Erato-sara Clade of Heliconius Butterflies

We retrieved the 21 chromosome alignments from the original data set (Edelman et al. 2019) following the same steps when we retrieved and measured the key characteristics of chr11 for the simulations (command lines are provided in the Supplementary Information). For each chromosome, we ran the stepwise non-overlapping window analysis with an initial window size of 64 kb, then made a series of pairwise comparisons by comparing the starting window size (e.g., 64 kb) to a window size of half that length (e.g., 32 kb). We generated individual gene trees for both window sizes using IQ-TREE2 (Minh et al. 2020) with the following command: iqtree2 -s path_to_fasta -blmin 1/window_size -bb 1000, where -s refers to the alignment file and -bb sets the number of UFBoot replicates. Then, we calculated the AIC for each window size by summing up the log-likelihoods and number of free parameters across windows following the original formula (Akaike 1974). When the number of unique sequences was less than four, we ran IQ-TREE2 without bootstrapping to save computational time. We continued to halve the window size (e.g., 32 and 16 kb, 16 and 8 kb, and so on) until it reached 125 bp, which cannot be divided by 2. Lastly, we summarized the topology distributions from the best window sizes (i.e., window size with the best AIC score for each chromosome) and compared our results with those from Edelman et al. (2019).

### Great Apes

We also ran the stepwise non-overlapping window approach on genome alignments of great apes, consisting of four species: *Homo sapiens* (hg38; human), *Pan troglodytes* (panTro4; chimpanzee), *Gorilla gorilla* (gorGor3; gorilla), and *Pongo pygmaeus* (ponAbe2; orangutan). First, we downloaded the compressed MAF file for each chromosome from the UCSC Genome Browser (https://hgdownload.soe.ucsc.edu/goldenPath/hg38/multiz20way), resulting in 25 chromosome (22 autosomal, X, Y, and mitochondrial) alignments. Then, we uncompressed the files and converted them to FASTA using PHAST v1.5 (Hubisz et al. 2011) with the following commands:

$ msa_view {i}.maf -i MAF -m –G 1 > {i}.fa

$ msa_view -l hg38,gorGor3,panTro4,ponAbe2 {i}.fa > greatapes_{i}.fa

where i represents the chromosome number (or name). We ran the stepwise non-overlapping window analysis on each chromosome following the same steps and parameters from the *Heliconius* analyses detailed above.

## Results

### Simulated Data Sets

**Figure 1.**
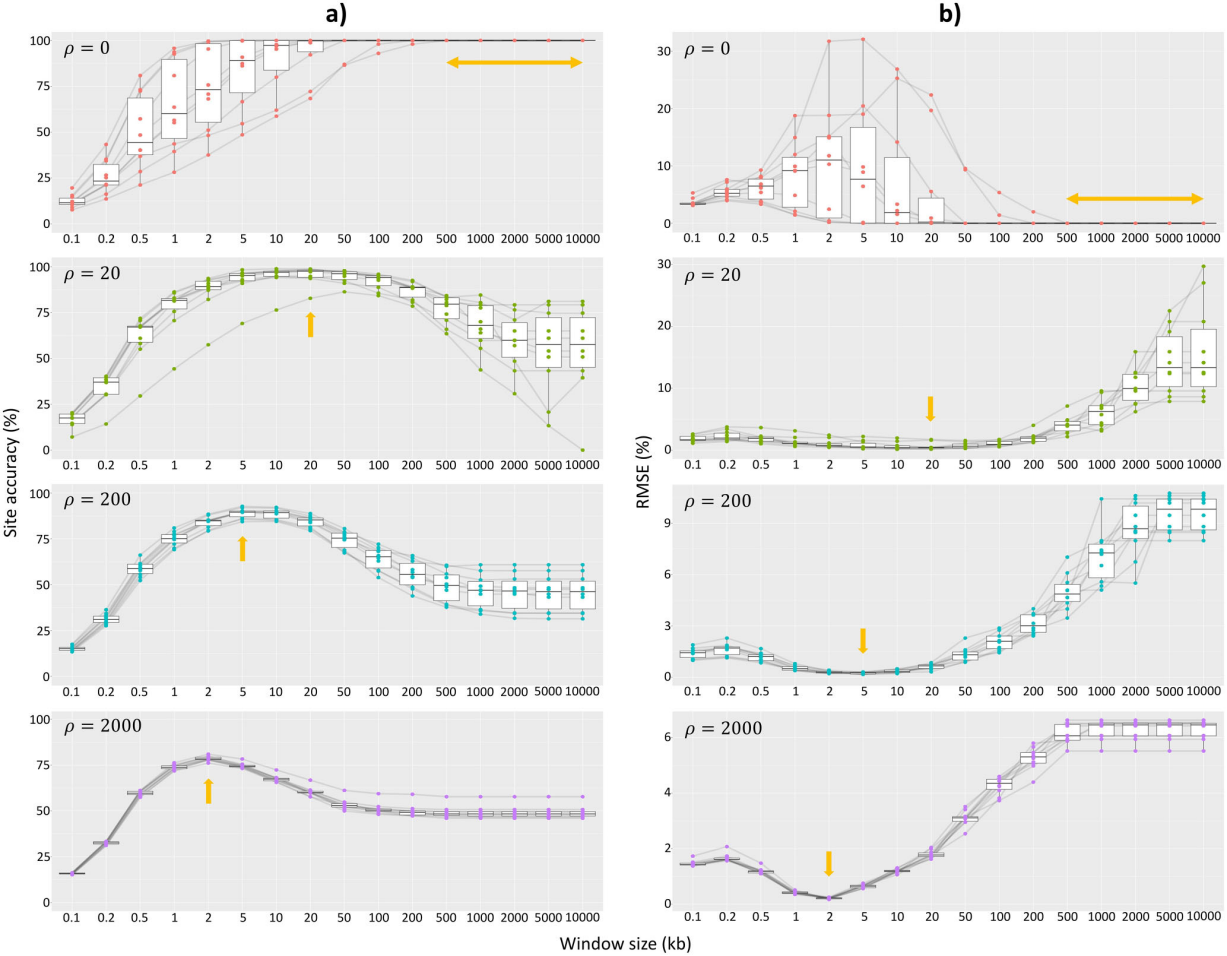
a) Site accuracy and b) RMSE of non-overlapping windows on simulated chromosomes with medium ILS level. Light orange arrows show window size(s) with the highest average site accuracy and lowest average RMSE across 10 replicates. Each dot represents an individual result. Black lines connect results from the same simulated alignment.

**Figure 2.**
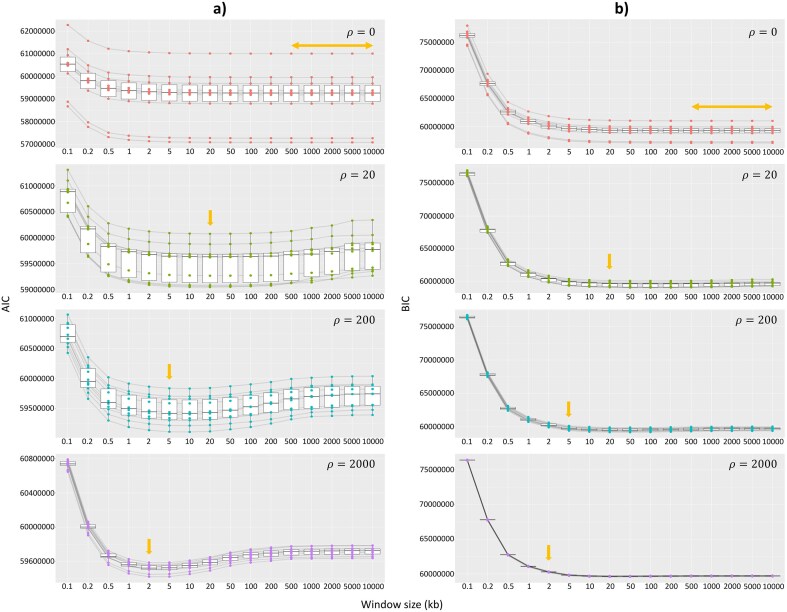
Correlation between the a) AIC and b) BIC with window sizes on simulated chromosomes with medium ILS level. Light orange arrows show window size(s) with the highest average site accuracy and lowest average RMSE from Figure 1. Each box represents different recombination rates. Each dot represents an individual result. Black lines connect results from the same simulated alignment.

Summary statistics of 120 simulated data sets are shown in Supplementary Table S3. We found that the degree of ILS had no meaningful effect on the results (Figs. 1 and 2, Supplementary Figs. S2–S20), so we focus on the results from the medium ILS simulations here (which most closely mimic the empirical data sets from Edelman et al. 2019) and present the rest in the Supplementary Information. Figure 1 shows that the best window sizes (i.e., window sizes with the highest site accuracy and/or lowest RMSE) become shorter as the recombination rate increases. This matches our expectation that the true locus lengths from the simulations would become shorter as the recombination rate increases due to more frequent topology switching, resulting in shorter best window sizes when running non-overlapping window analysis as they better reflect those topology breakpoints compared with the longer windows. For instance, the best window sizes for *ρ* = 0, 20, 200, and 2000 across 10 replicates are ≥50, 20, 5, and 2 kb, respectively (Fig. 1). Running an additional analysis with zero introgression rate and a medium level of ILS shows that when the recombination rate is not zero, the best window sizes are ∼50% smaller as the recombination rate increases by 10× (e.g., the best window sizes for *ρ* = 20 are 10–50 kb, whereas the best window sizes for *ρ* = 200 are 5–10 kb; Supplementary Fig. S21).

The best window sizes for each simulation (i.e., window sizes with the highest site accuracy and/or lowest RMSE) are generally longer than the average true locus length from ms when we consider both the tree topology and branch lengths of the simulated loci (Supplementary Fig. S22), but smaller than the average locus length when consecutive loci with the same topology are merged except when *ρ* = 2000 (Supplementary Fig. S23). This may reflect a compromise in selecting one window size that performs well across all loci of different lengths—long enough to accurately infer the local tree topology, but also short enough to minimize the information loss from concatenation. When the recombination rate is very high, this trade-off, coupled with very frequent topology switching, might lead to the maximum site accuracy achievable by non-overlapping window method to be ∼75% (Fig. 1). Note that the window size with the highest site accuracy does not always have the lowest RMSE (Supplementary Figs. S22 and S23). Thus, depending on the purpose of the analysis, we might want to use site accuracy (i.e., if we are interested in both the gene tree topologies and their locations along the genome) or RMSE as our criterion (i.e., if we are only interested in the distribution of the gene tree topologies regardless of their genomic positions) to choose the best window size.

Moreover, when the recombination rate is not zero, the site accuracies for longer window sizes tend to converge toward 50% (Fig. 1), because approximately 50% of the sites for each simulated alignment were simulated from the same topology, with other topologies represented in far smaller proportions. As more and more windows were concatenated, the site accuracy converges to 50% because this dominant topology is the one recovered by larger windows, to the exclusion of the other minor topologies. Interestingly, there is one simulation at *ρ* = 20 where the site accuracy reaches almost zero when using 10 Mb window (i.e., a fully concatenated analysis; Fig. 1). For this simulation, the most common topology simulated by ms groups species 5 and 6 together as the sister group of a clade formed by species 3 and 4 (i.e., ((1,2),((5,6),(3,4)));), accounting for a total of 25.8% of the sites in the chromosome. However, the (5,6) grouping is only supported by 41.6% of the sites overall, whereas the remaining sites were simulated from other topologies that often place species 5 and 6 far apart on the tree. This conflicting signal likely misleads the concatenation method—something that is well known to occur in related situations (Kubatko and Degnan 2007; Matsen and Steel 2007). For this simulated chromosome, the ML tree topology that is recovered when concatenating all windows places species 6 as the sister taxon of species 3, 4, and 5 (i.e., ((1,2),(6,(5,(3,4))));), with all splits having 100% UFBoot support. Remarkably, this topology was simulated for just 0.01% of the sites in the chromosome. This closely matches the findings from previous studies, which show that concatenating loci with differing coalescent histories can lead to the inference of highly supported trees (i.e., trees with high bootstrap support) that differ from the species tree (Kubatko and Degnan 2007).

To assess the potential utility of the AIC and BIC, we compared both with the site accuracy and RMSE measured from the simulated chromosomes. The AIC frequently picks the most accurate window size according to both criteria, but the BIC does not (Fig. 2, Supplementary Fig. S22, Supplementary Table S3). Although both the AIC and BIC seem to generally correlate with the two measures of accuracy (Supplementary Figs. S11 and S12), the BIC performs poorly in predicting accuracy, particularly when the recombination rate is very high (*ρ* = 2000) (Fig. 2b, Supplementary Fig. S13), leading to on average 18.5% site accuracy loss and 1.5% RMSE increase compared with the most accurate window size if we always choose window size with the lowest BIC (Supplementary Fig. S13b, d). This trend is also shown on the simulated data set without introgression, where the BIC prefers concatenation (i.e., 10 Mb window) when the most accurate window size ranges between 2 and 5 kb (Supplementary Fig. S21). On the other hand, the AIC is a good predictor of both site accuracy and RMSE (Fig. 2a, Supplementary Fig. S13). Similar to the two measures of accuracy, the AIC tends to select a window size that is somewhat larger than the peak of the simulated window size distribution when windows are defined by differences in topology *and* branch lengths, and somewhat smaller than the peak of the simulated window size distribution when windows are defined by differences in topology only (Supplementary Figs. S22 and S23). Specifically, the AIC often chooses the most accurate window size according to both the site accuracy and RMSE, with the highest average site accuracy loss of 0.25% and average RMSE gain of ∼0.1% when *ρ* = 200 if we always choose window size with the lowest AIC (Supplementary Fig. S13a, c). These results are consistent across data sets with different degrees of ILS (Supplementary Figs. S2–S8, S14–S20) and one without introgression event (Supplementary Fig. S21), and thus we propose that the AIC can be used to select the best window size on empirical alignments.

### Empirical Data Sets

In order to address the issue of missing data in empirical alignments, we designed a stepwise non-overlapping window approach that compares two window sizes at a time and includes only windows that can be analyzed using both window sizes. This approach enabled us to use shorter windows while ensuring the alignment consistency between the two window sizes, which is crucial when comparing the AIC scores. All results for the empirical data sets were derived from the stepwise non-overlapping window method rather than the common non-overlapping windows approach.

### Erato-sara Clade of Heliconius Butterflies

Using the stepwise non-overlapping window approach, we found that the sex chromosome and the 10 longest autosomal chromosomes in *erato-sara* clade of *Heliconius*’ genomes have a best window size of 250 bp, whereas the AIC of other chromosomes was still declining after reaching 125 bp with much smaller delta AIC scores than the previous step (500 bp vs. 250 bp) (Supplementary Tables S4 and S5), suggesting that the best window size for these chromosomes is at most 125 bp. Moreover, we also found that the selection of window sizes affects the distribution of the tree topologies recovered from the group (Supplementary Tables S6 and S7). For example, the topology distribution of chr11 only includes <10 unique topologies and is dominated by Tree 1 using 64 kb windows, but becomes much more variable as the window sizes become smaller due to gene tree estimation error (Supplementary Fig. S24).

**Figure 3.**
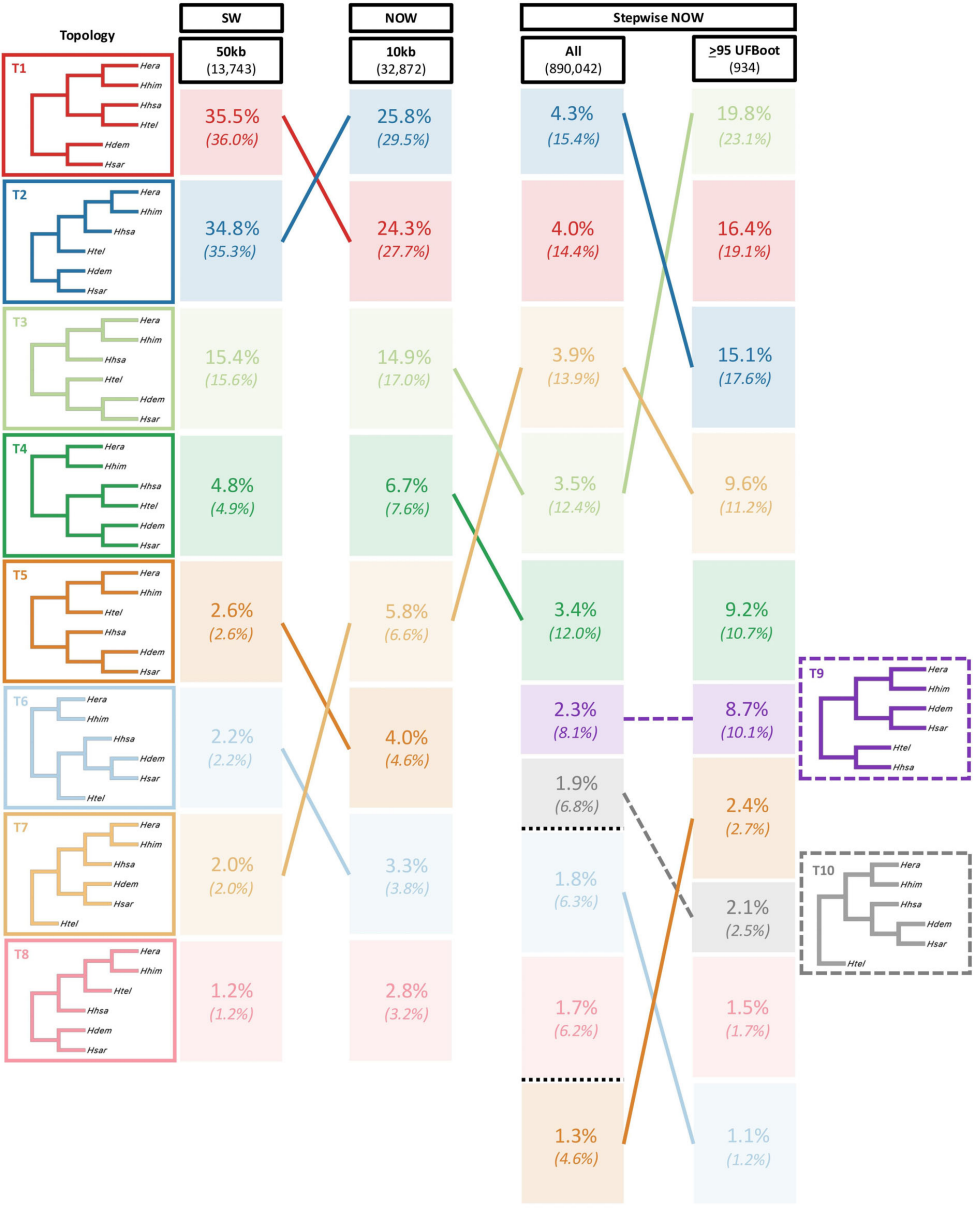
Comparison of topology frequency of the most common tree topologies from *erato-sara* clade of *Heliconius*’ genomes between 50 kb sliding windows (SW) and 10 kb non-overlapping windows (NOW) from Edelman et al. (2019), as well as stepwise non-overlapping windows (stepwise NOW) using the best window size for each chromosome (Supplementary Table S4). Topologies are rooted with *H. melpomene* (not shown) as the outgroup. The number under each method reflects the total number of windows. Dashed lines refer to additional topologies that are not present in the original eight, whereas black dotted lines refer to other topologies not listed. Numbers in brackets show the normalized value for each distribution, rounded to one decimal place, so the total might not be exactly 100%. Topologies are visualized using FigTree v1.4.4 (http://tree.bio.ed.ac.uk/software/figtree) without branch lengths.

Figure 3 shows the most common tree topologies recovered from the sliding window (first column) and non-overlapping window analyses (second column) in Edelman et al. (2019) as well as the stepwise non-overlapping windows (third and fourth columns) from our analyses, where the best window size was selected for each chromosome using the AIC (Supplementary Table S4). The stepwise non-overlapping window approach recovers a much broader distribution of tree topologies, with the most frequently recovered five topologies making up only 19.1% of all gene trees, most likely due to gene tree estimation error introduced by very short window sizes (Fig. 3 [third column], Supplementary Table S8). This is also reflected by the number of consecutive windows that recover the same topology, which is mostly one (Supplementary Fig. S25). When considering only the well-supported tree topologies (i.e., trees with ≥95 average UFBoot support), the most frequent 10 topologies account for 85.9% of all well-supported tree topologies (Fig. 3 [fourth column], Supplementary Table S9). Using this approach, Tree 3 is the most common topology, closely followed by Trees 1 and 2 from the original study (in which Tree 2 is considered to be the species tree). Interestingly, gene trees with the Tree 3 topology have a lower average sum of internal branch lengths across all gene trees (0.0709 substitutions per site) compared with Tree 1 (0.0813 substitutions per site) and Tree 2 (0.0772 substitutions per site), but a slightly higher average number of parsimony informative sites (14.2) compared with the other two topologies (13.4 for Tree 1 and 14.0 for Tree 2). This analysis also finds two additional topologies, Tree 9 and Tree 10, that, respectively, represent 8.7% and 2.1% of the well-supported trees.

### Great Apes

Supplementary Table S10 shows the results of the stepwise non-overlapping window analyses for the great apes. The best window size for most chromosomes is either 500 bp or 1 kb, except for the mitochondrial DNA (best window size of 4 kb and all windows recover the same topology; Supplementary Fig. S26), and the Y chromosome (best window size of 500 bp, but noting that this alignment included just 2.24% of the initial alignment, reflecting the presence of all-gapped taxa in most of the windows).

**Figure 4.**
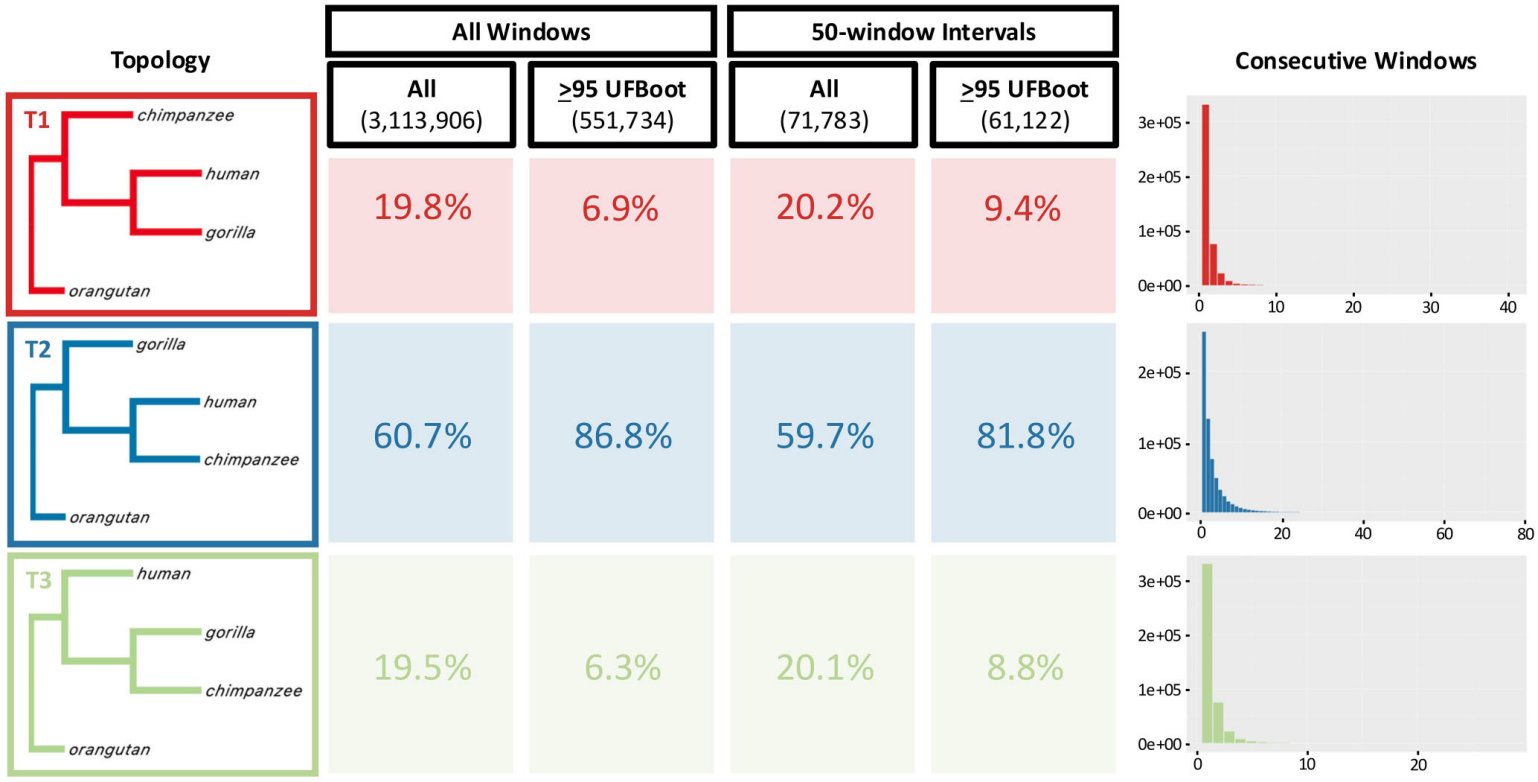
Comparison of unrooted tree topology frequency from great apes’ genomes when including all windows (first and second columns) and gene trees with a minimum of 50-window interval (third and fourth columns) based on stepwise non-overlapping windows using the best window size for each chromosome (Supplementary Table S10). The rightmost panels show the number of consecutive windows that recovers each topology from stepwise non-overlapping windows considering all gene trees. The number under each method reflects the total number of windows. Topologies are visualized using FigTree v1.4.4 (http://tree.bio.ed.ac.uk/software/figtree) without branch lengths.

The distribution of tree topologies from the stepwise non-overlapping windows follows the expected pattern, in which the (major) topology that groups human and chimpanzee is the most common, followed by a roughly equal proportion of the other two (minor) topologies. For example, if we count all gene trees based on the best window size for each chromosome in Supplementary Table S10, the ratio between the major topology and the two minor topologies is roughly 60.7:19.8:19.5 (Supplementary Table S11 [left], Fig. 4 [first column]). If we consider only highly supported gene trees with ≥95 average UFBoot support, the proportions change to 86.8:6.9:6.3 (Supplementary Table S11 [right], Fig. 4 [second column]). However, both of these approaches may not reflect the underlying distribution of tree topologies, as including all trees might introduce gene tree estimation error while highly supported trees might bias toward one or a few topologies (see Discussion). Moreover, Supplementary Figure S27 and Figure 4 (rightmost panel) show that the number of consecutive windows that recover the same topology is mostly 20-windows long or less by considering all trees. If we are only interested in the gene tree topology, we can define a non-recombining block (or window) as consecutive windows that share the same topology. As one non-recombining block might be partitioned into several windows when running the non-overlapping window analysis, we might want to space out the gene trees being analyzed so that the gene tree topology distribution reflects the “true” number of non-recombining windows of the alignment. Separating the trees by at least 50 windows, which is likely to be sufficient to ensure that each tree is independent of each other, shows that the proportions approach an asymptote of approximately 60:20:20 when considering all trees and 82:9:9 for highly supported ones (Fig. 4, Supplementary Fig. S28 [third and fourth columns]). This might suggest that the effect of double (or multiple) counting of one non-recombing block is less apparent when considering all gene trees (proportion of major topology changed from 60.7% to 59.7%, Fig. 4) compared with the highly supported trees (proportion of major topology changed from 86.8% to 81.8%, Fig. 4).

**Figure 5.**
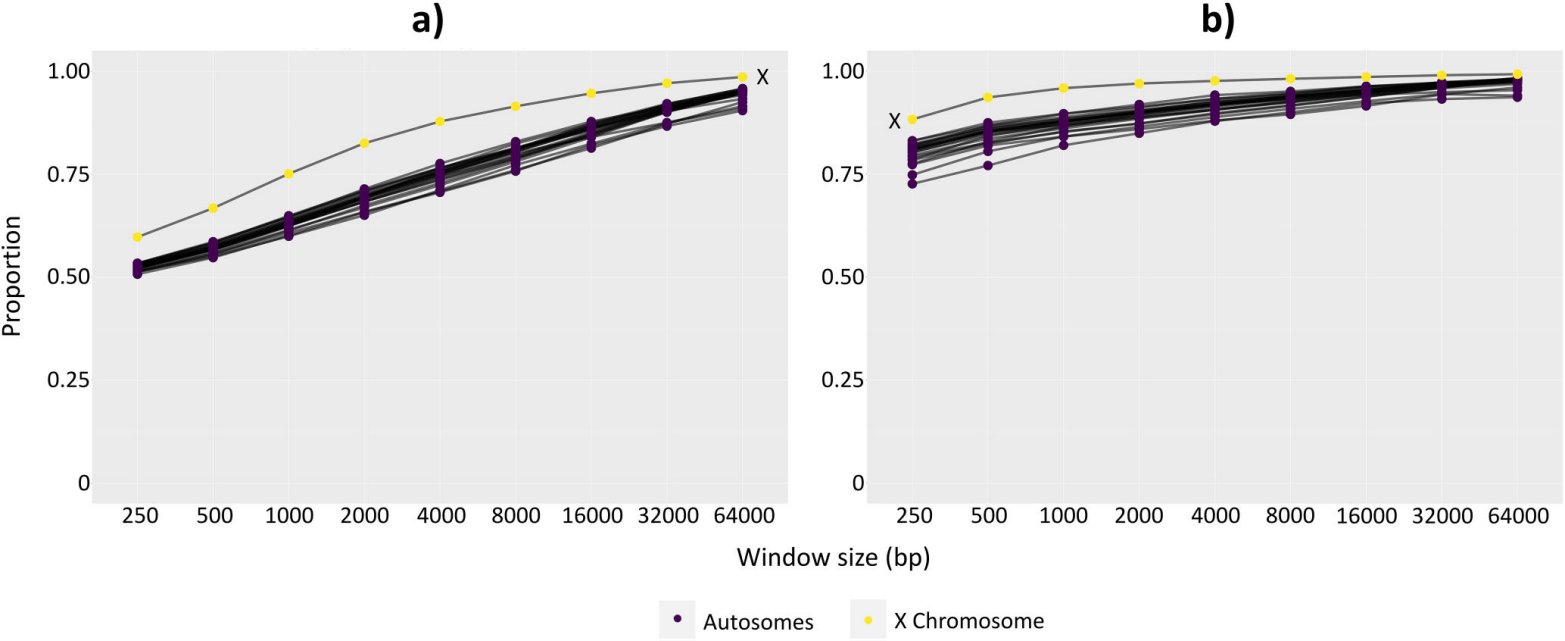
Proportion of major topology of great apes (grouping human and chimpanzee) from stepwise non-overlapping windows based on a) all gene trees and b) gene trees with ≥95 average UFBoot support. Black lines connect the same chromosome across window sizes.

We further investigated the impact of window size on empirical tree distributions by comparing the distribution of tree topologies across the whole range of window sizes (Fig. 5). At small window sizes, such proportions are likely to be highly affected by gene tree estimation error, whereas at large window sizes, they are likely to be affected by concatenation (the inclusion of one or more recombination breakpoints in a single locus). Figure 5 shows the genome-wide proportion of the major topology (grouping human and chimpanzee) calculated from all trees (Fig. 5a) and highly supported trees (i.e., trees with ≥95 average UFBoot support, Fig. 5b) across all window sizes. Consistent with the effect of gene tree estimation error, the inferred proportion of the major topology is much lower across all trees than it is for highly supported trees, particularly at smaller window sizes (compare Fig. 5a with Fig. 5b, also Supplementary Figs. S29 and S30). On the other hand, the proportion of the major topology shows a monotonic increase in most chromosomes as the window size increases, which follows the effect of concatenation (see Discussion for more details).

## Discussion

The non-overlapping window method is commonly used in phylogenomic studies to infer local histories along a set of genomes. However, the selection of the window size is often arbitrary and might not closely reflect the optimal size to extract non-recombining regions, affecting the inference of tree topologies in different genomic regions. In order to address this problem, we simulated chromosomes that mimic the empirical alignments of *erato*-*sara* clade of *Heliconius* butterflies (Edelman et al. 2019) with varying recombination rates. We then analyzed these simulated data sets using different non-overlapping window sizes and showed that the AIC is a strong correlate of two different measures of accuracy: the proportion of sites in the genome assigned to the correct tree topology (i.e., site accuracy) and the distribution of tree topologies recovered from the analysis (i.e., RMSE). The BIC, on the other hand, tends to overpenalize short window sizes by considering the length of the window alignment. As a result, it always chooses much longer window sizes than the most accurate ones (Fig. 1, Supplementary Figs. S22 and S23).

Recombination events can produce neighboring windows that share the same topology but differ in branch lengths. In principle, the AIC should be able to detect such differences given enough data, and the results from our simulations suggest that this is the case. When the recombination rate is low and the average window size from the simulated data is long, the AIC selects a window size that falls between the average window size based on both the topologies and branch lengths, and the average window size based on the topologies alone (Supplementary Figs. S22 and S23). When the recombination rate is high and the average simulated window size is short, the AIC selects a window size that closely matches the average window size based on the topologies alone (Supplementary Fig. S23) but is much longer than the average window size based on both the topologies and branch lengths (Supplementary Fig. S22). This suggests that the AIC has a limited ability to distinguish branch length variation between neighboring windows with identical topologies when the windows themselves are short. This limitation may be an important consideration when choosing a window size for analyses that rely on accurate branch length reconstructions, such as demographic inference.

We developed a stepwise approach that allowed us to use the AIC to select the optimal window size for empirical data sets. Applying this method to genomes of the *erato-sara* clade of *Heliconius* from Edelman et al. (2019), the best window size for each chromosome ranges from ≤125 to 250 bp (Supplementary Table S4), smaller than the predicted linkage blocks in *H. erato* by Tobler et al. (2005) and Counterman et al. (2010). As our data set does not only include *H. erato* but also other closely related species, our estimates should include older recombination events that have occurred during the evolution of the group, resulting in c-genes (non-recombining blocks) that are shorter than the linkage blocks in any single species if there is a recombination ratchet (i.e., the reduction of c-gene size when the number of taxa increases due to more recombination events on the tree, see Springer and Gatesy 2016, 2018). These window sizes are also vastly shorter than those used in the original study (Edelman et al. 2019), which used 10 and 50 kb windows in different analyses. Somewhat reassuringly, the different window-based methods that we and others have applied to this data set do not significantly change the most common topologies inferred across the genomes of the group, particularly when considering the highly supported trees (Fig. 3, Supplementary Table S7). Thus, if the focus is simply on identifying the most common topologies without considering the proportion of each topology (e.g., as the input data for MAST [Wong et al. 2024]), then the exact choice of window size may not significantly affect the set of topologies recovered. However, if the *distribution* (or proportions) of the topologies and/or their locations along the genome are the focus (e.g., in the estimation of the extent of recombination and hybridization, reconstruction of recombination-based linkage maps, or association between genotype and phenotype), then the selection of window size matters when using non-overlapping windows (Supplementary Tables S6 and S7, Supplementary Fig. S24). In addition, it should be noted that the data sets from Edelman et al. (2019) are haploid representations of the *Heliconius* genomes. Allelic phasing has been proposed to improve the estimates of tree topology (Andermann et al. 2019; but see also Kates et al. 2018), whereas random phasing (or the use of haploid consensus sequences) might bias the inference of both gene trees and species trees (Huang et al. 2022). Although we did not test for the effect of phasing on our method, we hypothesize that the best window size for phased genome alignments might be smaller than the one for haploid consensus sequences as the stepwise approach would account for the allelic variations derived from phasing. Thus, careful consideration needs to be exercised when interpreting the results from stepwise non-overlapping windows on unphased genomes, particularly the inference of recombination rate based on the best window size selected by the AIC.

For great apes, the best window size for each chromosome ranges from 500 bp to 1 kb (and 4 kb for the mitochondrial genome) (Supplementary Table S10). These are around 4× longer than the best window sizes from *Heliconius* (≤125–250 bp), likely reflecting the lower average recombination rates in great apes (∼1 cM/Mb) (The International HapMap Consortium 2007; Stevison et al. 2016) compared with *Heliconius* butterflies (∼6 cM/Mb) (Jiggins et al. 2005; Tobler et al. 2005). Previous work comprising human, chimpanzee, and orangutan has suggested that the length of genomic regions that recover the species tree topology (grouping human and chimpanzee) ranged from 900 bp to 7.8 kb, whereas genomic fragments with alternative genealogies can be very short (<100 bp; Hobolth et al. 2011). On the other hand, Springer and Gatesy (2016) estimate that the mean c-genes for human, chimpanzee, and gorilla to be around ∼109 bp. Our results are 5×–10× larger than these estimates, likely due to the lack of phylogenetic signal in shorter windows.

The best window size of 4 kb for the mitochondrial genome might be counterintuitive at first—this 16 kb genome is inherited as a single locus, and recombination is vanishingly rare (though likely not completely absent [Kraytsberg et al. 2004]), suggesting that a single window should be recovered, not four. However, all four of the 4 kb windows for the mitochondrial genome have the same topology, and it is simply the branch lengths that differ (Supplementary Fig. S26), reflecting the expected capability of the AIC to consider both the tree topology and branch lengths when selecting a window size. The selection of four windows instead of one likely occurs because of model misspecification, such that allowing for four windows with identical topologies and slightly different branch lengths is a better fit to the data than having two windows or a single window. Similar observations were made by Richards et al. (2018), who showed that the inferred tree topologies of different regions of the mitochondrial genome often differ because of model misspecification.

Our analyses highlight the challenges of estimating the distribution of gene tree topologies from whole genome alignments. In the analysis of great apes, there are only three possible unrooted tree topologies. Figure 5 shows that the frequency of the species tree topology (which in this case is also the major topology, grouping human and chimpanzee) is just 50% when using 250 bp windows, but increases to almost 100% when using 64 kb windows. This dramatic change occurs because of two competing effects: gene tree estimation error and concatenation. Gene tree estimation error in shorter windows is caused by a lack of phylogenetic signal and tends to push the proportion of the topologies toward a more random distribution (in this case, a frequency of 33.3% for the species tree topology as there are only three possible topologies). We also observed similar patterns with *erato-sara* clade of *Heliconius* butterflies, where the five most common topologies derived from *all* 125–250 bp windows only account for 19.1% of all windows (Fig. 3). At chromosomal level, the effect of gene tree estimation error is evident on the sex chromosome, where Tree 2 (the species tree topology that should dominate the sex chromosome [Edelman et al. 2019]) only accounts for 10.14% of all gene trees (Supplementary Table S8) or 35.85% of well-supported trees (Supplementary Table S9) of chromosome 21. On the other hand, longer windows are more likely to include recombination breakpoints (i.e., concatenation of two or more non-recombining blocks). As a result, the gene trees estimated from these windows often reflect the average phylogenetic signals across the regions, which can bias the inference toward one or a few topologies depending on the model parameters (Roch and Steel 2015). In great apes, this favors the species tree topology, pushing its proportion toward 100% (Fig. 5).

Previous studies have sometimes attempted to ameliorate the effects of gene tree estimation error by analyzing “highly supported” trees, for example, those with average bootstrap support ≥95%. However, high bootstrap value does not confirm that the inferred tree is correct, but rather indicates that the branching events are consistently supported by the data. Moreover, Bryant and Hahn (2020) pointed out that this approach will bias the distribution toward gene trees with longer internal branches. For butterflies of the *erato-sara* clade of *Heliconius*, filtering out gene trees with average bootstrap support <95% from the stepwise approach resulted in Tree 3 (an inversion topology found on chromosome 2 [Edelman et al. 2019]) as the most common topology (Fig. 3). However, it is likely that this distribution is significantly affected by gene tree estimation error, as gene trees with the Tree 1 and Tree 2 topologies tend to have longer average internal branch lengths than the gene trees with Tree 3 topology. This might show the limitation of the non-overlapping window approach when the window size is very short, and further confirms that bootstrap values should not be used to measure the “correctness” of the tree. On the other hand, for great apes, the estimated frequency of tree grouping human and chimpanzee is far higher across almost every chromosome (Fig. 5b) than when we analyzed all trees (Fig. 5a), and this effect is repeated on the trees from the optimal window sizes (Fig. 4). Nevertheless, these approaches might allow us to estimate a plausible range within which the frequency of the major tree topology lies. Using the optimal window size for each chromosome of the great apes’ genomes, spaced 50 windows apart to prevent multiple counting of the same non-recombining blocks (Fig. 4), the complete distribution of tree topologies suggests that the major topology has a frequency of 60% (with the other two possible topologies having a frequency of 20% each). This estimate can be considered a lower bound because it would be driven down by gene tree estimation error in short windows. When we analyzed only those trees from the same set with high bootstrap support, the frequency became 82% for the major topology (and 9% each for the alternative topologies). This estimate represents an upper bound because it is biased upwards by the longer internal branches in the loci that match the species tree topology. The truth is likely to be somewhere in-between, which agrees closely with many previous estimates of this frequency, ranging from 60% to 77% depending on the methods being used (Ebersberger et al. 2007; Vanderpool et al. 2020; Wong et al. 2024).

A number of approaches can be used to further refine the gene trees estimated from the methods we present here, particularly when the best window size is very short and ML methods are limited in their ability to infer a reliable gene tree for each window. Such methods include ARG reconstruction using Espalier (Rasmussen and Guo 2023) and the multi-tree mixture model from MAST (Wong et al. 2024). All of these methods take different approaches to the same end—the estimation of the topology underlying every site in the alignment—but critically, they combine information from windows across the genome alignments to improve the estimation of the gene tree for each window. Most also benefit considerably from the initial selection of the optimal window size and the estimate of the tree associated with each window. In conclusion, our AIC-based stepwise non-overlapping method provides a less arbitrary way to select the best window size given the alignments. One key limitation of this method is that only a single window size is selected for each aligned chromosome. Future work will focus on estimating variable window sizes across the genome by dividing long windows and/or joining together two neighboring windows.

## Supplementary Material

syaf053_Supplemental_File

## Data Availability

Summary tables from the simulations and R scripts necessary to reproduce the analyses reported in this study can be accessed through the Dryad link: https://doi.org/10.5061/dryad.jdfn2z3ng.

## Supplementary Materials

Data available from the Dryad Digital Repository: https://doi.org/10.5061/dryad.jdfn2z3ng
